# Ectopic Liver Tissue Encountered Incidentally During a Laparoscopic Cholecystectomy: A Case Report

**DOI:** 10.7759/cureus.42220

**Published:** 2023-07-20

**Authors:** Gagandeep Singh Arora, Shivam Kalra

**Affiliations:** 1 Department of General Surgery, Government Medical College, Patiala, IND; 2 Department of Internal Medicine, Trident Medical Center, North Charleston, USA; 3 Department of Internal Medicine, Dayanand Medical College and Hospital, Ludhiana, IND

**Keywords:** hepatic choristoma, gallbladder anomalies, hepatic tissue, aberrant liver tissue, case report, laparoscopic cholecystectomy, gallbladder, ectopic liver

## Abstract

A 60-year-old male patient who presented with right upper quadrant (RUQ) pain was diagnosed with acute cholecystitis after an ultrasound of the abdomen revealed multiple gallstones, gallbladder wall thickening, pericholecystic fluid, and a positive sonographic Murphy sign. The patient was admitted, administered IV fluids, antibiotics, and pain relief, and scheduled for laparoscopic cholecystectomy. During surgery, an incidental finding of ectopic liver tissue attached to the gallbladder was noted. Histopathology confirmed the presence of chronic cholecystitis and multifaceted cholesterol stones. Normal liver tissue was noted in the ectopic mass. Ectopic liver tissue is defined as liver tissue located outside the main liver parenchyma and is usually asymptomatic. They are usually detected at the time of autopsies, incidentally during surgeries, or during imaging done for other etiologies. They can occur at various sites in the body. Ectopic liver tissue can cause potential complications such as hepatocellular carcinoma and torsion, and in the event that they are incidentally detected, it is advised to remove them. The case report highlights the importance of dealing with incidental findings during laparoscopic cholecystectomy and creating awareness about it.

## Introduction

Variations in anatomy often lead to incidental discoveries during surgical procedures [1]. Ectopic liver tissue is defined as liver tissue located outside the liver with no direct connection to the liver [2]. It is often an incidental finding during surgeries, autopsies, or diagnostic imaging [3]. With a prevalence rate of approximately 0.28% in laparoscopic procedures, these anomalies are usually asymptomatic and often go unnoticed until they present as soft-tissue masses during routine medical procedures [2].

This report presents a unique case of an ectopic liver mass discovered during laparoscopic cholecystectomy, one of the most frequently performed surgical procedures worldwide [4]. Because it is a rare and complex discovery, it is important to understand not only the immediate surgical implications but also the potential long-term effects on patient health and quality of life [5]. When found, it is recommended that these should be resected since they are potentially susceptible to conditions such as hepatocellular carcinoma [5].

Furthermore, this report highlights the embryology of ectopic liver tissue, providing insight into the mechanisms behind its occurrence in varied body sites, including the spleen, umbilicus, vena cava, heart, and lung [2]. Ectopic liver tissue is associated with complications, and our case report highlights the importance of understanding the vascular supply for efficient surgical intervention [6]. Our case of an ectopic liver mass was not detected on ultrasound but was revealed during laparoscopic cholecystectomy. This emphasizes the importance of awareness about this rare condition.

## Case presentation

The patient is a 60-year-old male with a past medical history of hypertension and on a daily 5 mg regimen of amlodipine who presented with acute right upper quadrant (RUQ) pain that started suddenly. The pain was severe, with an intensity of 8/10, and radiated to the back. The patient reported he had been having episodes of RUQ pain in the past, but this time, the pain persisted and only partially subsided with antacid use.

**Figure 1:**
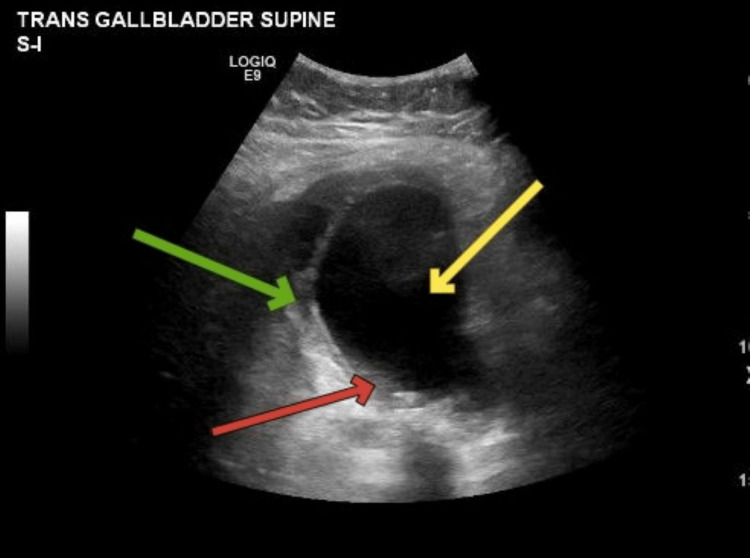
Ultrasound showing the gallbladder Ultrasound of the gallbladder showing a yellow arrow pointing at the distended gallbladder, a green arrow pointing at the gallbladder wall thickening with some pericholecystic fluid, and a red arrow pointing at the gallbladder stones.

Physical exam was significant only for a positive Murphy sign. On initial evaluation, vital signs were stable. Laboratory investigations revealed slight leukocytosis, with a white blood cell count of 11,800 cells per microliter (normal range: 4,500-11,000 per microliter of blood). All other lab investigations, including hematocrit, renal function tests, liver function tests, amylase, and lipase levels, were within normal limits. An ultrasound of the upper abdomen was conducted which revealed multiple gallstones. The ultrasound also showed gallbladder wall thickening, pericholecystic fluid, and a positive sonographic Murphy sign which were consistent with a diagnosis of acute cholecystitis. The diameter of the common bile duct was normal, and there was no dilation of the intrahepatic biliary radicles. The liver parenchymal echogenicity was homogeneous (Figure 1).

**Figure 2:**
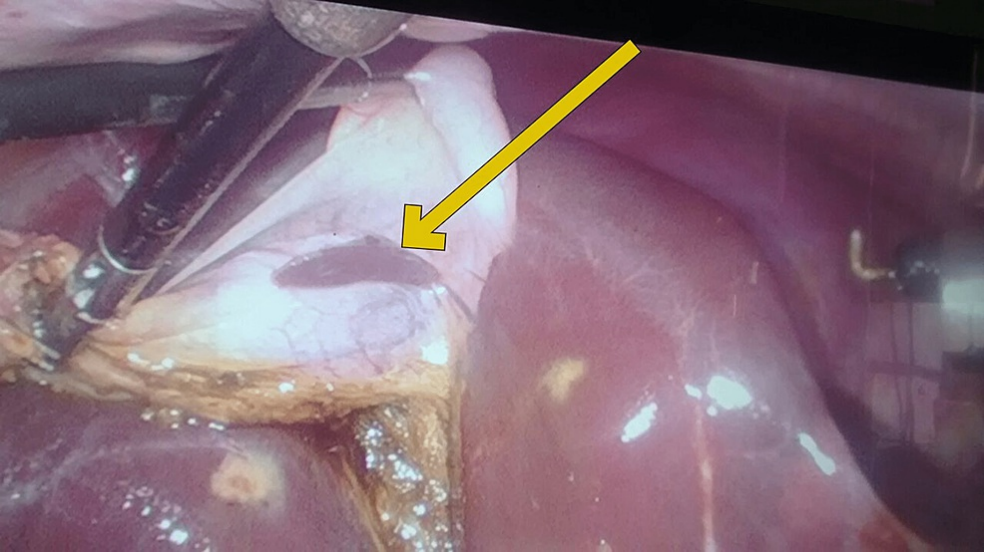
Laparoscopic view during cholecystectomy The medial surface of the gallbladder showing a yellow arrow pointing toward ectopic liver tissue.

The patient was admitted, hydrated with IV fluids, and administered antibiotics and pain relief. We decided to take the patient up for laparoscopic cholecystectomy the coming morning after informed consent was taken, including consent for an open procedure in case a conversion was required. While in the operating room, pneumoperitoneum was established with ease, and the gallbladder was easily retracted. The gallbladder was inflamed, but there were not many adhesions to the omentum. After dissection of the hepatocystic triangle, the cystic duct and artery were easily identified and ligated. During the dissection of the gallbladder from the liver bed, we realized that there was a small tissue with its own blood supply present on the gallbladder. The color and texture of the tissue were the same as that of the liver (Figure 2).

**Figure 3:**
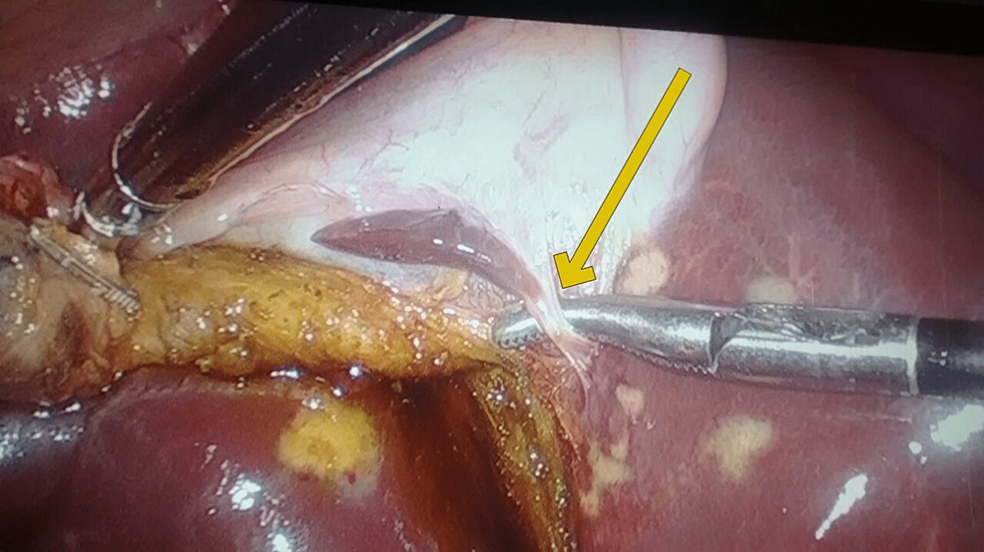
Laparoscopic view during cholecystectomy showing blood supply of ectopic tissue The medial surface of the gallbladder with a yellow arrow pointing toward ectopic liver tissue with its own independent blood supply

**Figure 4:**
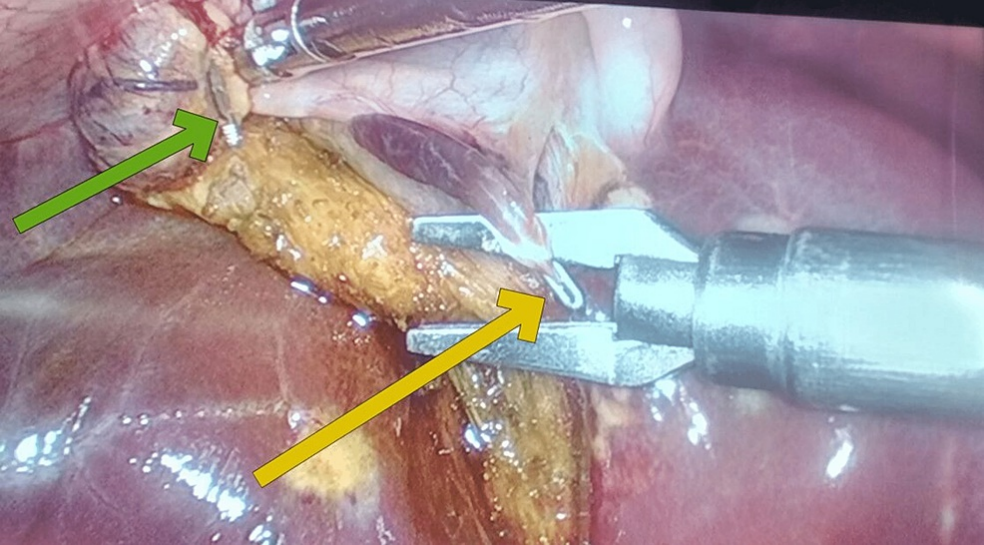
Laparoscopic view during cholecystectomy showing a clip being applied to the blood supply of ectopic tissue A laparoscopic view of the gallbladder while doing resection from the liver bed with a yellow arrow pointing toward the clipped blood supply of the ectopic liver and a green arrow pointing at the clip applied and the ligated cystic duct.

We identified it to be a remanent ectopic liver tissue. We were careful in our dissection of the gallbladder from the liver bed taking care not to avulse the blood supply of the ectopic tissue. Therefore, we dissected the feeding blood vessel and applied another clip to it to prevent postoperative bleeding. Hence, an incidental finding of a small liver-like tissue adherent to the medial surface of the gallbladder was noted (Figures 3, 4).

The gallbladder, along with the ectopic liver tissue, was sent for histopathology. Postoperatively, the patient was informed about the incidental findings. The patient's postoperative stay was uneventful. He was later discharged with advice to avoid fatty foods for a couple of weeks. Histopathology results later revealed chronic cholecystitis and multifaceted cholesterol stones and confirmed the presence of normal liver tissue in the ectopic mass adherent to the gallbladder.

## Discussion

Laparoscopic cholecystectomy is the most commonly performed surgical procedure [4] and is also the most likely to have incidental findings [1]. The incidence of incidental findings in abdominal surgery is estimated to be 1.1% [1]. When dealing with incidental findings, it is important to consider not only the immediate surgical implications but also the potential long-term effect it can have on the patient's health and quality of life [1]. In our case, the ectopic liver mass was an incidental finding.

Ectopic liver tissue is defined as liver tissue located outside the liver that has no connection with the main liver parenchyma [7].

Diagnosis

They are usually asymptomatic and detected incidentally during surgery or autopsy, but they can be seen when a soft-tissue mass arises from the gallbladder wall on abdominal ultrasound or CT scan [2]. Histopathology usually confirms the diagnosis of liver tissue [7].

Incidence and diagnosis

In a study of 5,500 autopsies, only 0.05% revealed the presence of ectopic liver tissue, with only three cases specifically showing attachment to the gallbladder wall [2]. In another study analyzing 1,060 laparoscopic procedures, researchers identified ectopic liver tissue attached to the gallbladder wall in only three patients, indicating a prevalence rate of 0.28% [2].

Location of ectopic hepatic tissue

An ectopic liver can occur in varied sites of the body. They have been found in the spleen, umbilicus, vena cava, heart, and even the lungs [2]. There has even been a case report where an ectopic liver was seen herniating through the diaphragm into the mediastinum, giving an appearance of right atrial myxoma on an echocardiogram [8]. Huang et al. reported a case where an ectopic liver mass was seen in the submucosal layer of the stomach, and it was removed with the help of an endoscope [7]. Ectopic liver tissue can have its own mesentery and resembles normal liver histologically [2].

Collan et al. classified ectopic liver depending upon location into four categories [9]. The first type corresponds to an accessory liver lobe, which possesses the ability to grow to a significant size and is connected to the liver through a stalk [9]. The second type is characterized by a small accessory liver lobe, typically weighing between 10 to 30 grams, which is attached to the liver [9]. The third type encompasses ectopic liver tissue, which is situated outside the liver without any direct connection to it [9]. It is often found attached to the gallbladder or intra-abdominal ligaments [9]. Lastly, the fourth type involves microscopic ectopic liver tissue occasionally detected within the wall of the gallbladder [9].

Embryology of ectopic liver tissue

Various theories have emerged to elucidate the mechanisms underlying the occurrence of ectopic liver in different locations. These theories encompass concepts such as the development of accessory liver lobes, migration or displacement of liver bud, dorsal budding of hepatic tissue, entrapment of hepatocyte-destined mesenchymal cells, and the entrapment of cell nests in the foregut region [7].

Complications of ectopic liver tissue

The presence of ectopic liver tissue is frequently associated with inadequate biliary drainage and reduced blood supply, which has been suggested as a possible explanation for the heightened susceptibility to hepatocellular carcinoma in individuals with this condition [5]. Multiple studies have documented instances of hepatocellular carcinoma linked to ectopic liver tissue with varying degrees of susceptibility depending on the specific location of the ectopic liver [2]. Ectopic liver tissue can undergo pathological changes similar to the main liver, such as fatty change, hemosiderosis, cholestasis, or cirrhosis [2]. On rare occasions, ectopic livers have been reported to cause recurrent abdominal pain, attributed to torsion [10].

Importance of vascular supply

Knowledge of the vascular supply becomes important for the removal of ectopic tissue.** **Bal et al. described a mass with a unique blood supply [6]. Our case too had its own blood supply. Accurate identification of the vascular pedicle of ectopic liver tissue, which typically arises directly from the liver substance or the cystic artery, is crucial prior to gallbladder dissection from the liver bed to prevent potential rupture or tearing of vascular structures derived from the liver substance due to excessive traction during removal [2]. Three vascular supply patterns have been described: an artery from the cystic artery, a vascular pedicle from the liver parenchyma, and vascular structures in a mesentery connecting the hepatic site to the ectopic liver tissue [2].

Surgical intervention is necessary for identifying the vascular supply and preventing bleeding [3]. Caution is advised with direct vascular supply from the liver substance [3]. Biliary drainage is often incomplete [3]. The ectopic liver can affect the biliary tree as it develops from the hepatic diverticulum, which also gives rise to the gallbladder so it can potentially cause biliary obstruction or other complications, but the specific effect on the biliary tree may vary depending on the location and size of the ectopic liver [3]. The removal of a known asymptomatic ectopic liver must be considered because of the potential for torsion and because of the increased risk for malignancy [11]. When found serendipitously intraoperatively, it is always prudent to remove the ectopic liver tissue [12].

Lundy et al. suggested that laparoscopic management of the ectopic liver is safe and effective and may offer advantages such as reduced postoperative pain, shorter hospital stay, and faster recovery compared to open surgery [3]. They also noted that laparoscopic management of the ectopic liver required careful preoperative planning and imaging to ensure complete resection of the ectopic liver and preservation of surrounding structures [3].

Associations with other congenital abnormalities

Park et al. reported a case of umbilical liver and biliary atresia [13]. Furthermore, Shapiro et al. reported a heterotopic supradiaphragmatic liver in an individual with congenital cardiac anomalies, further supporting the association between congenital abnormalities and the presence of ectopic livers [14].

Our case

Our case had a small ectopic liver mass attached to the medial surface of the gallbladder. The mass was not seen on ultrasound. In our case as well, there was a similar case described by Bal et al. [6]. The mass had its own blood supply, and we did not take a risk and decided to proceed with dissecting the pedicle. In our institute, we strive for optimal hemostasis in order to prevent the need for drain placement. Therefore, we applied a clip to the pedicle. We can very well imagine that in case the ectopic liver was large, it would have caused anatomic distortion and difficulty in the dissection of the hepatocystic triangle which could have complicated the procedure. Thus, awareness about ectopic liver found incidentally during laparoscopy is important.

## Conclusions

Ectopic livers are often diagnosed at autopsy, incidentally during surgery, and had imaging done for other issues. Ectopic liver tissue is a rare developmental anomaly and occurs along with other congenital abnormalities. The unique intraoperative finding of ectopic liver tissue adherent to the gallbladder underscores the importance of a meticulous surgical approach. Ectopic livers are associated with an increased propensity to develop hepatocellular carcinoma, abdominal pain due to torsion, and other complications. Therefore, it is always prudent to remove incidentally found ectopic livers during laparoscopy. Ectopic livers can potentially complicate the procedure if they are larger in size. Hence, it is important to create awareness about such findings.
